# Continuous Plasma Exchange With Dialysis for Severe Sepsis: Case Series of a Novel Blood Purification Method

**DOI:** 10.7759/cureus.12495

**Published:** 2021-01-05

**Authors:** Kasumi Satoh, Manabu Okuyama, Yasuhito Irie, Toshiharu Kitamura, Hajime Nakae

**Affiliations:** 1 Department of Emergency and Critical Care Medicine, Akita University Graduate School of Medicine, Akita, JPN

**Keywords:** severe sepsis, plasma exchange, dialysis, intensive care unit

## Abstract

Sepsis is associated with life-threatening organ dysfunction. Drastic treatment methods such as antimicrobials and surgical control have been used to manage sepsis. However, there are currently no other sepsis-specific treatments capable of improving mortality rates. Plasma exchange (PE) for the removal of toxic substances and the replacement of consumed bioprotective proteins has been advocated as a potential treatment for sepsis. Although the evidence for the efficacy of PE for sepsis is quite limited, in a recent finding, sepsis patients treated with PE showed improvement in fluid balance and organ damage. Continuous PE with dialysis (cPED), which is a modified version of PE, is a novel blood purification method. cPED is a combination of selective PE and hemodialysis and operates slowly in a simple circuit that can potentially provide powerful supportive care for patients with multiple organ failure. In this report, we present two cases of treatment with cPED in patients with severe sepsis and organ damage. Both patients were discharged alive without any adverse events from cPED. cPED improved fluid balance as well as laboratory parameters that had reflected multiple organ failure. This suggests a possible reduction in side effects such as leakage of bio-essential proteins and citric acid reactions to large doses of fresh frozen plasma. The clinical course of these two patients may be useful for setting outcomes in future clinical studies regarding the effectiveness of cPED for severe sepsis.

## Introduction

Sepsis is associated with life-threatening organ dysfunction caused by abnormalities in the host's response to the infection [1]. Drastic treatments for sepsis include antimicrobials and surgical control. However, there are currently no other sepsis-specific treatments that have the ability to improve mortality rates among affected patients. Severe sepsis is a devastating illness that has been reported to kill up to half of the affected patients [2]. Host responses due to cytokine release, inflammatory cell attraction, and endothelial dysfunction strongly influence the severity of sepsis; for example, endothelial dysfunction, which can lead to disseminated intravascular coagulation (DIC), causing multi-organ failure due to extensive coagulation of the microvascular system [3]. Deficiency of protein C and ADAMTS13 has also been found to occur in sepsis [3]. Therefore, plasma exchange (PE) to remove toxic substances and replace consumed bioprotective proteins has been advocated as a treatment modality for sepsis [3]. However, evidence for the efficacy of PE for treating sepsis is limited. Evidence published so far includes case reports and case series, retrospective single-center observational studies, and meta-analyses of randomized controlled studies with an undeniable high degree of bias, with the American Society for Apheresis (ASFA) recommending that PE, as a treatment for sepsis with organ damage, is allowed on a case-by-case basis [4,5]. The effect of PE treatment in patients with severe sepsis was examined in a propensity score-matched PE group of 40 patients and a control group of 40 patients, and it showed improvements in 28-day mortality, fluid balance, and organ damage at 48 hours in the PE group [6]. A prospective study by Knaup et al. reported that hemodynamic improvements were observed when PE was introduced in sepsis treatment [7].

Continuous PE with dialysis (cPED), which is a modified version of PE, is a novel blood purification method. cPED is a combination of selective PE and hemodialysis and operates slowly in a simple circuit that can potentially provide powerful supportive care for patients with multiple organ failure [8]. In principle, compared to conventional PE, cPED is associated with a reduction in adverse events, economic benefits, and compatibility with hemodynamic instability [8]. The cPED study for acute liver failure showed improvement in serum total protein (TP) level, fibrinogen (Fib) level, prothrombin time-international normalized ratio (PT-INR), creatinine (Cre) level, along with an absence of hypoalbuminemia [8].

This report is a case series study detailing the clinical course of two patients who were treated with cPED for severe sepsis in the intensive care unit (ICU).

## Case presentation

**Figure 1:**
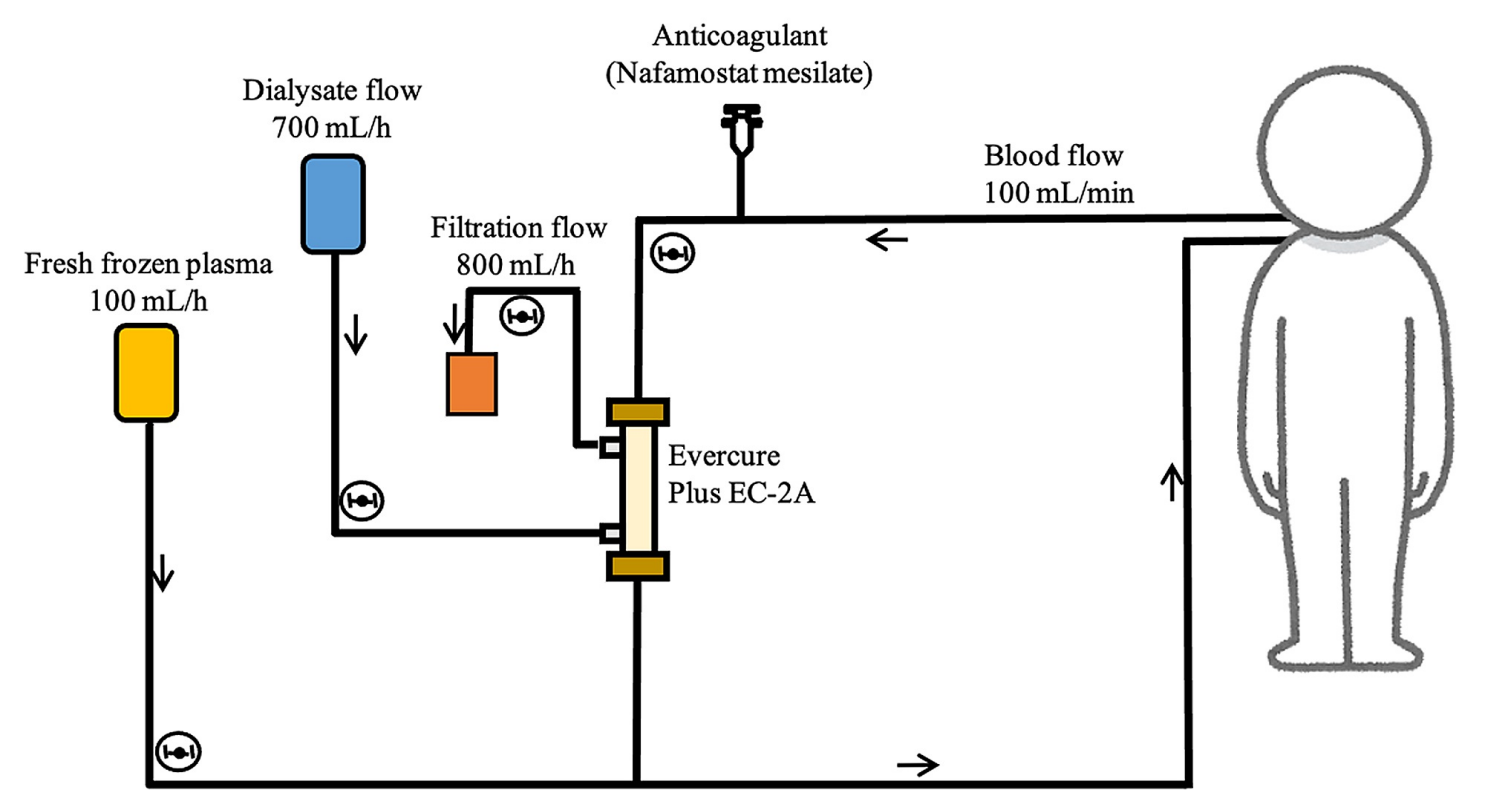
The circuit of continuous plasma exchange with dialysis The image outlines a simple circuit for selective plasma exchange and continuous hemodialysis on a single console

**Figure 2:**
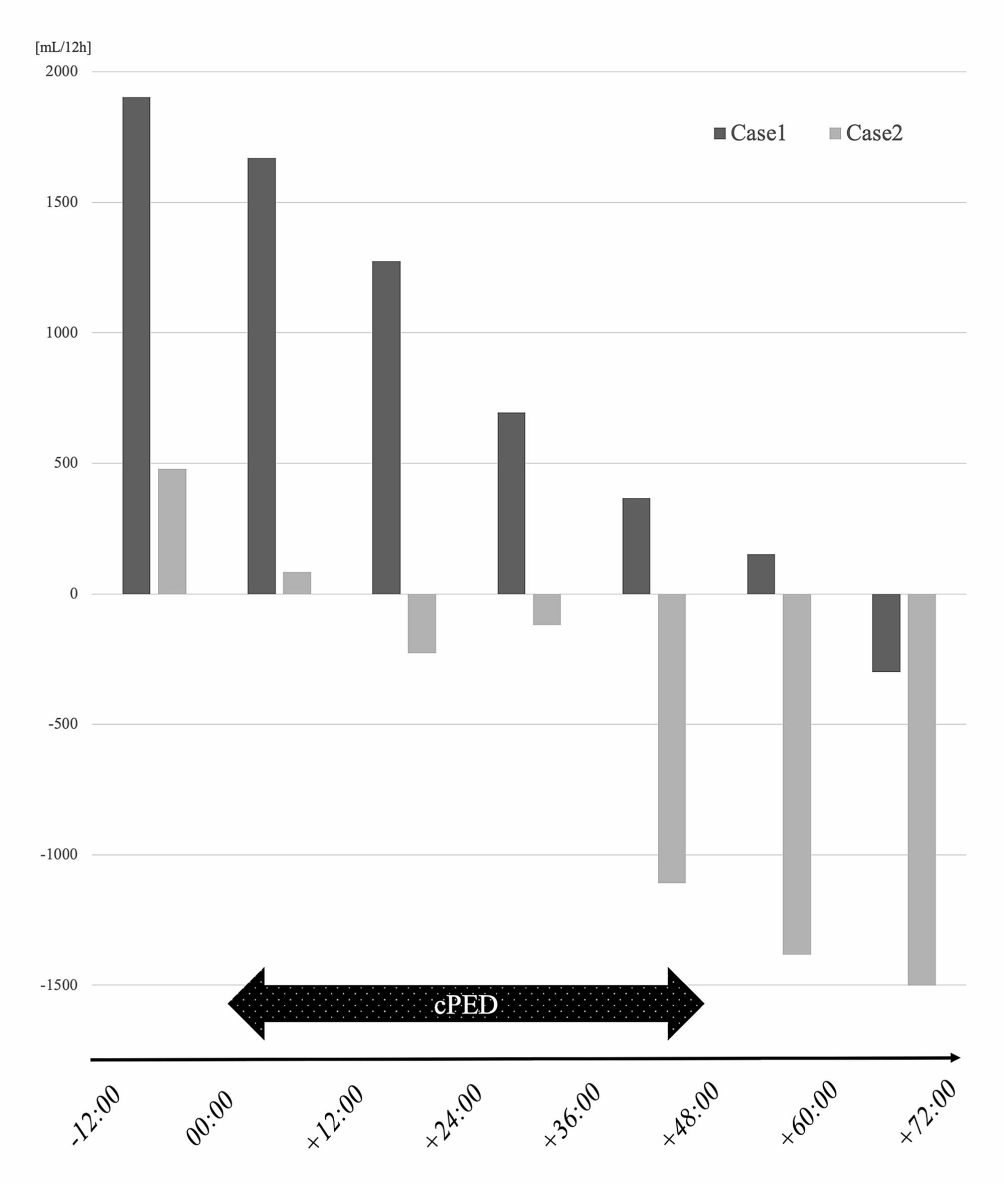
Total fluid volume recorded every 12 hours from 12 hours before to 72 hours after the start of continuous plasma exchange with dialysis In both cases, it was apparently observed that the total fluid balance moved to the decreasing side after the initiation of continuous plasma exchange with dialysis cPED: continuous plasma exchange with dialysis

Our report discusses the cases of two septic patients with concomitant multiple organ damage treated in the ICU (Table 1). Laboratory data collected within 24 hours from the start of cPED were considered as pre-cPED data, and data collected within 24 hours after the end of cPED were considered as post-cPED data. The circuit of the cPED is shown in Figure 1. Both patients had coagulopathy, including disseminated intravascular coagulation syndrome, renal impairment, and hepatic impairment. cPED began approximately one day after ICU admission; one session lasting approximately 48 hours was performed. Improvements in various laboratory parameters for kidney, liver, and coagulation were observed after cPED. Albumin levels did not drop, and electrolytes were stable. Both patients progressed well and were able to leave the ICU alive. Figure 2 shows the total fluid balance recorded every 12 hours from 12 hours before cPED till 72 hours after the start of cPED. After the initiation of cPED, the fluid balance was found to move towards the dry side.

**Table 1:** Case overview and summary of laboratory data before and after treatment Laboratory data collected within 24 hours from the start of continuous plasma exchange with dialysis (cPED) were considered as pre-cPED data, and data collected within 24 hours after the end of cPED were considered as post-cPED data. Both were cases of severe sepsis; however, some improvement in laboratory data was observed after implementation of cPED, and the patients were discharged with survival Abbreviations (units): PLT: platelet (×10,000/μL); PT: prothrombin time; APTT: activated partial thromboplastin time (seconds); Fib: fibrinogen (μg/mL); FDP: fibrin/fibrinogen degradation products (μg/mL); TP: total protein (g/dL); Alb: albumin (g/dL); AST: aspartate aminotransferase (U/L); ALT: alanine aminotransferase (U/L); T-bil: total bilirubin (mg/dL); BUN: blood urea nitrogen (mg/dL); Cre: creatinine (mg/dL); Na: sodium (mEq/L); K: potassium (mEq/L); Ca: calcium (mg/dL); APACHE II: Acute Physiology and Chronic Health Evaluation II; SOFA: Sequential Organ Failure Assessment

Case	Age (years)	Sex	APACHE II score	SOFA score	Outcome	Focus	Causative organism		PLT	PT %	APTT	Fib	AT-3	D-dimer	FDP	TP	Alb	AST	ALT	T-bil	BUN	Cre	Na	K	Ca
Case 1	56	Male	20	7	Survival	Lung	Legionella	Pre-cPED	5.6	39.6	90.7	654	57.5	116.5	236.9	4.3	2	4,323	2,280	1.4	44.7	2.97	127	3.8	9.5
								Post-cPED	11.5	89.9	51.1	668	49.2	13.18	29.7	4.6	1.8	252	315	0.8	31	1.88	133	3.6	10.8
Case 2	46	Male	22	10	Survival	Bloodstream	Candida	Pre-cPED	13.3	24.7	121	166	48.7	42.9	85.9	3.9	2.7	1,000	905	2.3	32.8	1.96	137	4.6	9.4
								Post-cPED	7.7	75.6	52.9	377	64.1	3.92	8.9	5.2	2.8	82	99	2.2	19.3	0.79	140	3.8	10.6

Case 1

**Figure 3:**
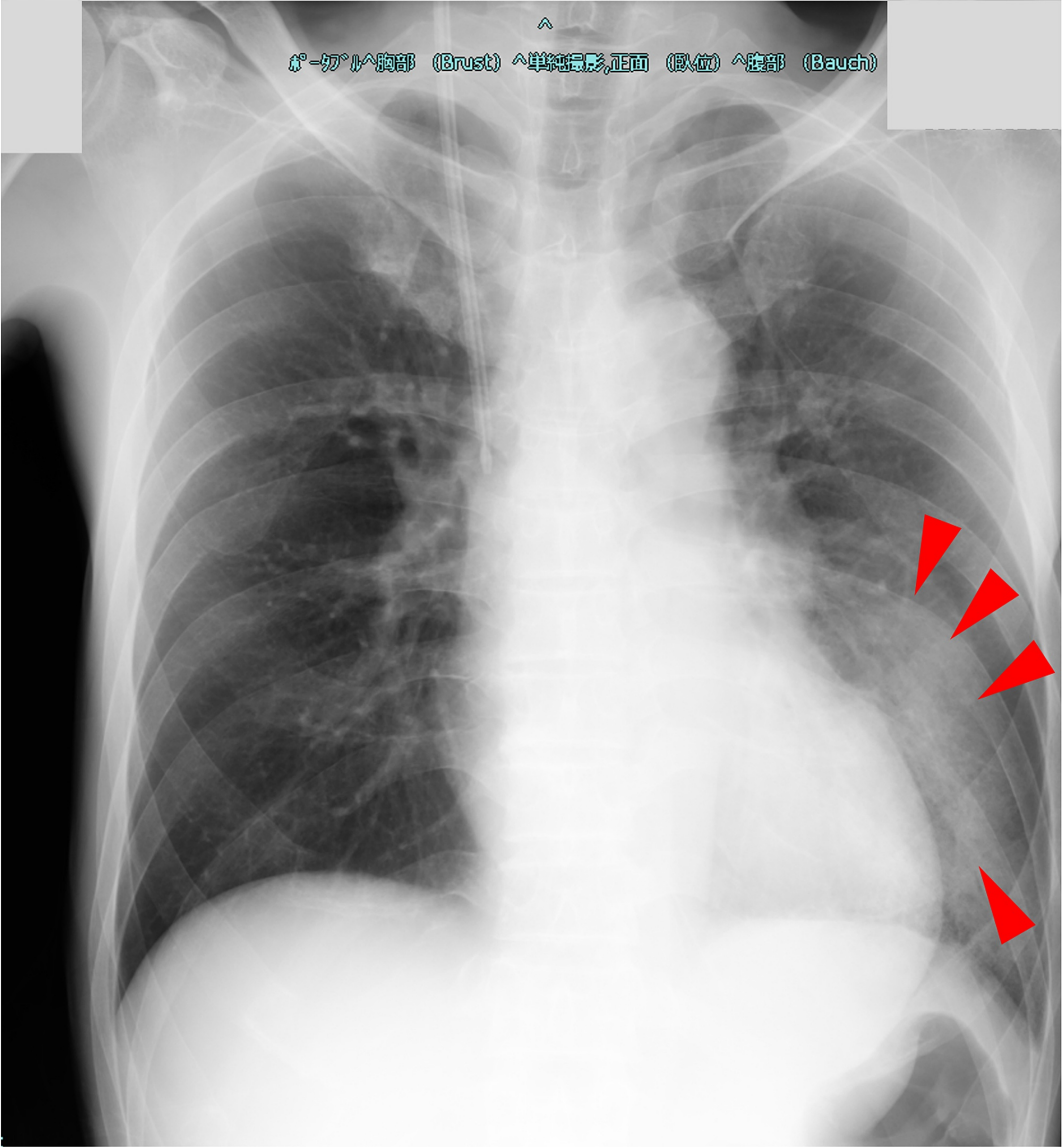
Chest X-ray taken on the first day in the intensive care unit Chest X-ray showed widespread consolidation and air-alveologram in the left lung (arrowheads) suggesting severe pneumonia

A 56-year-old man (weight approximately 50 kg) with no medical history was admitted to the emergency department with shaking chills, loss of appetite, and difficulty in mobility, which had begun five days earlier. He was soon transferred to the ICU with a diagnosis of severe pneumonia. During our examination, he was alert, and his blood pressure, heart rate, cardiac rhythm, respiratory rate, oxygen saturation, and body temperature were 173/101 mmHg, 130 beats/min (atrial fibrillation), 42 breaths/min, 97% [high-flow nasal cannula (HFNC), 30 L/min, 30%], and 39.0 °C, respectively. Arterial blood gas findings were as follows: pH of 7.45, PaO_2_ of 110 (HFNC, 30 L/min, 30%), PaCO_2_ of 20, HCO_3_ of 16.9, and lactate of 13 mg/dL. The white blood cell (WBC) count was 13,400/μL, hemoglobin (Hb) level was 14.4 g/dL, and platelet (PLT) count was 92,000/μL. The blood urea nitrogen (BUN) level was 59.4 mg/dL, the Cre level was 4.09 mg/dL, alanine aminotransferase (ALT) level was 4,599, aspartate aminotransferase (AST) level was 12,743 U/L, total bilirubin (T-bil) level was 2.1 mg/dL, sodium (Na) level 128 mEq/L, potassium level was 3.6 mEq/L, and creatinine kinase (CK) level was 16,092 IU/L. The C-reactive protein (CRP) and procalcitonin (PCT) levels were both abnormal at 25.65 mg/dL and 106.3 ng/dL, respectively. Coagulation abnormalities were obvious, with an activated partial thromboplastin time (APTT) of 44.4 seconds, a prothrombin time (PT) of only 32.0% of the normal range, 307.9 μg/mL of fibrin/fibrinogen degradation products (FDP), D-dimer level of 155.0 μg/mL, a fibrinogen level of 667.0 mg/dL, and an antithrombin III (AT-III) level of only 45.6% of the normal range. The Sequential Organ Failure Assessment (SOFA) score [9] was 7 points, and the Japanese Association for Acute Medicine Disseminated Intravascular Coagulation (JAAM DIC) diagnostic criteria score [10] was 6 points. Chest X-ray findings showing widespread consolidation and air-alveologram in the left lung (Figure 3), along with the detection of urinary Legionella antigen, led to a diagnosis of severe pneumonia due to Legionella. In the ICU, the patient received 500 mg intravenous levofloxacin every 24 hours, landiolol, recombinant human soluble thrombomodulin, and antithrombin gamma. We started continuous hemodiafiltration (CHDF) on the first day of ICU and switched to cPED the next day. After one session of cPED (48 hours), CHDF was reintroduced and terminated on day nine of the ICU stay. The patient was transferred to the general ward on the 11th day in a stable condition without oxygen administration.

Case 2

A 46-year-old man (weight approximately 50 kg) with Crohn's disease from adolescence and short bowel syndrome was admitted to the gastroenterology general ward due to fever. He received total parenteral nutrition and developed a catheter-related bloodstream infection. On the third day of hospitalization, he was transferred to our ICU due to hypotension and multiple organ impairment. On examination, he was alert, and his blood pressure, heart rate, respiratory rate, oxygen saturation, and body temperature were 74/44 mmHg, 130 beats/min, 16 breaths/min, 92% (under ambient air), and 37.5 °C, respectively. Arterial blood gas findings were as follows: pH of 7.44; PaO_2_ of 75; PaCO_2_ of 37; HCO_3_ of 25.1, and lactate of 32 mg/dL. His lactate level was elevated, and he developed septic shock. The WBC count was 13,000/μL, the Hb level was 13.5 g/dL, and the PLT count was 142,000/μL. The BUN level was 40.5 mg/dL, the Cre level was 3.78 mg/dL, the ALT level was 834 U/L, the AST level was 12,743 U/L, the T-bil level was 2.3 mg/dL, the Na level was 130 mEq/L, and the K level 4.8 mEq/L. The CRP and PCT levels were both abnormal at 6.00 mg/dL and 5.24 ng/dL, respectively. Coagulation abnormalities were also present, with an APTT of 55.0 seconds, a PT of only 28.8% of the normal range, FDP of 103.0 μg/mL, a D-dimer level of 52.05 μg/mL, a fibrinogen level of 222.0 mg/dL, and an AT-III level of only 38.7% of the normal range. β-d-glucan was within the normal limit of 4.77 pg/mL. The SOFA score [9] was 10 points, and the JAAM DIC diagnostic criteria score [10] was 5 points. In the ICU, he received 0.5 g meropenem intravenous every 12 hours, 100 mg micafungin intravenous every 24 hours, recombinant human soluble thrombomodulin, antithrombin gamma, and 200 mg/day of hydrocortisone intravenously; norepinephrine was initiated at approximately 0.15 μg/kg/min. We started CHDF on the first day of ICU and switched to cPED the next day. After one session of cPED, CHDF was reintroduced and terminated on day seven of the ICU. Candida parapsilosis was later detected in his blood culture. The patient was retransferred to the general gastrointestinal ward on the eighth day in a stable condition.

## Discussion

Based on our findings, the introduction of cPED in the acute phase of severe sepsis has the potential to favorably affect multiple organ failure support and fluid balance regulation. Both patients had severe sepsis with hepatic impairment, renal impairment, and coagulopathy, and their condition improved after the introduction of cPED approximately one day after their admission to the ICU. No major adverse events due to cPED were observed.

cPED is a new blood purification method that simplifies plasma diafiltration (PDF), which utilizes a selective membrane plasma separator to perform PE while the dialysate is pumped outside the hollow fiber [8]. PDF has already been used for acute liver failure [11-13]. Continuous PDF, in which PDF is performed at a slower place in hemodynamically unstable patients, is used clinically [14]. However, PDF circuitry is complex and there is a risk of shortened column life and hemolysis. Therefore, cPED was designed to increase perfusion flow and perform without diluting fresh frozen plasma (FFP) [8]. The Evacure EC-2A plus®︎ selective plasma separation membrane used in cPED has a much smaller pore size (0.01 mm) compared to conventional plasma separation membranes (0.2-0.4 mm) [8]. The sieving coefficient for albumin is 0.3, which allows for the selective removal of low and medium molecular weight albumin-binding substances. The membrane has a fibrinogen sieving coefficient of zero, which effectively retains the essential substances [8]. In addition, the control of citrate concentration is difficult in conventional PE due to the high infusion volume of FFP [8]. In cPED, the pace of PE is slower, and hemodialysis is performed simultaneously, which, in principle, allows for more effective control of serum citrate levels [8]. The daily dose of FFP is 2,400 mL, which is economically advantageous compared to conventional PE. cPED is superior to conventional PE in terms of adverse event reduction, adaptability to hemodynamically unstable patients, and economy. In addition, cPED combines selective PE and hemodialysis, which can provide powerful supportive care for patients with multiple organ failure. Thus, cPED may be suitable for critically ill patients. cPED treatment in acute liver failure has been shown to improve liver damage parameters [8], and we are conducting a study on the efficacy of cPED in thrombotic microangiopathy (jRCTs022190024). However, this report is the first of its kind to investigate cPED treatment for sepsis.

After cPED, a clear improvement in coagulation parameters such as PT, APTT, Fib, FDP, and D-dimer was observed. cPED retains Fib, which is an advantage over conventional PE. However, AT-III is removed by selective PE in terms of molecular weight and tends to be low in the pathogenesis of sepsis; thus, AT-III was low in both cases and even decreased in Case 1, despite the fact that both patients were treated with AT-III supplement therapy. It seems that AT-III levels need to be carefully monitored during cPED administration. Albumin is less likely to decrease, and favorable control of Cre and Na was observed; no decrease in Ca, which is a surrogate indicator for the citric acid reaction, was observed. Our results suggest the potential of cPED as an organ supportive therapy and the mildness of adverse events; however, physicians should be cautious regarding the decrease in AT-III.

A retrospective observational study by Hadem et al. showed a significant decrease in net fluid balance within 12 hours after the first dose of conventional PE [15]. Although a simple comparison cannot be made because of the long duration of our cPED, which lasted 48 hours per session, we also observed a steady decrease in the fluid balance every 12 hours in our cPED-treated sepsis patients. Reducing fluid balance may be beneficial for vascular permeability, in addition to an improvement of oncotic pressure by isovolemic protein substitution [15]. Given the recent concerns about organ damage caused by excessive fluid infusion and the report of a 10% increase in the mortality rate for each 1-L increase in fluid balance in the first 72 hours of sepsis [16], this positive impact on fluid balance may have prognostic implications. We need to accumulate a higher number of sepsis cases and conduct further studies to conclusively determine the outcome of fluid balance changes due to the introduction of cPED.

## Conclusions

We reported two cases of cPED treatment in septic patients with multiple organ failure who were discharged from the ICU alive without any adverse events of cPED. We observed a trend of improved laboratory data and the absence of adverse effects after the introduction of cPED. After the initiation of cPED, we observed the possibility of a favorable change in fluid balance. The clinical course of these two cases has the potential to be useful in setting outcomes for future clinical studies regarding the effectiveness of cPED treatment for severe sepsis.
